# Tracing non-fungal eukaryotic diversity via shotgun metagenomes in the complex mudflat intertidal zones

**DOI:** 10.1128/msystems.00413-25

**Published:** 2025-06-12

**Authors:** He Han, Mengzhi Ji, Yan Li, Xiaofan Gong, Wen Song, Jiayin Zhou, Kai Ma, Yuqi Zhou, Xia Liu, Mengqi Wang, Yueyue Li, Qichao Tu

**Affiliations:** 1 Institute of Marine Science and Technology, Shandong University 520252, Qingdao, Shandong, China; 2 Qingdao Key Laboratory of Ocean Carbon Sequestration and Negative Emission Technology, Shandong University 520252, Qingdao, Shandong, China; CNRS Delegation Alpes, Lyon, Rhône-Alpes, France

**Keywords:** non-fungal eukaryotes, DNA fragments, community diversity, eukaryotic recovery, intertidal ecosystems, shotgun metagenomes, temporal patterns

## Abstract

Eukaryotes, both micro- and macro-, constitute the dominant component of Earth’s biosphere visible to the naked eye. Although relatively big in organismal size, tracing eukaryotic diversity in complex environments is not easy. For example, they may actively escape from sampling and be physically absent from the collected samples. In this study, we strived to recover non-fungal eukaryotic DNA sequences from typical shotgun metagenomes in the complex mudflat intertidal zones. Multiple recently developed approaches for identifying eukaryotic sequences from shotgun metagenomes were comparatively assessed. Considering the low overlap among different approaches, an integrative workflow was proposed. The integrative workflow was then used to recover the eukaryotic communities in complex intertidal sediments. The temporal dynamics of intertidal eukaryotic communities were investigated through a time-series sampling effort. Thirty-four non-fungal eukaryotic phyla were detected from 36 shotgun metagenomes. Clear temporal variation in relative abundance was observed for eukaryotic genera such as *Timema* and *Navicula*. Strong temporal turnover of intertidal eukaryotic communities was observed. By comparing to 18S rRNA gene amplicon sequencing, dramatically different community profiles were observed between these two approaches. However, the temporal patterns for intertidal eukaryotic communities recovered by both approaches were generally comparable. This study provides valuable technical insights into the recovery of non-fungal eukaryotic information from complex environments and demonstrates an alternative route for reusing the massive metagenomic data sets generated in the past and future.

**IMPORTANCE:**

Eukaryotes represent the dominant component visible to the naked eye and contribute to the primary biomass in the Earth’s biosphere. Yet, tracing the eukaryotic diversity in complex environments remains difficult, as they can actively move around and escape from sampling. Here, using the intertidal sediments as an example, we strived to retrieve non-fungal eukaryotic sequences from typical shotgun metagenomes. Compared to 18S rRNA gene amplicon sequencing, the shotgun metagenome-based approach resolved dramatically different eukaryotic community profiles, though comparable ecological patterns could be observed. This study paves an alternative way for utilizing shotgun metagenomic data to recover non-fungal eukaryotic information in complex environments, demonstrating significant potential for environmental monitoring and biodiversity investigations.

## INTRODUCTION

Eukaryotes, especially the ones visible to the naked eye, constitute the dominant component in the Earth’s biosphere. Although the number of eukaryotic species is far below that of prokaryotes, more than 8.7 million eukaryotic species are still estimated to exist on Earth (1, 2), including fungi, protozoa, plants, invertebrates, and vertebrates. Of these, fungi can usually be effectively profiled by universal primers targeting internally transcribed spacer, and sometimes 18S rRNA genes (3). In contrast, morphology-based approaches remain a major way to profile non-fungal eukaryotes. However, conventional identification and profiling of non-fungal eukaryotic communities relying on morphological observations via vision and microscopes are challenging and ineffective, especially for relatively small-sized eukaryotes. This is especially true now that skilled morphologists are becoming rarer in the era of genomics and molecular ecology (4). In addition, some large-sized eukaryotic organisms may actively escape from sampling and be physically absent from the samples. Therefore, sequencing-based approaches have become more widely used for non-fungal eukaryotic diversity investigations in complex natural ecosystems in recent years (5–7).

To date, multiple sequencing-based approaches have been developed to detect and profile non-fungal eukaryotic communities in complex environments. These include, but are not limited to, amplicon sequencing of marker genes (e.g., 18S rRNA genes and mitochondrion DNA) and shotgun metagenomic sequencing (8–12). Over the past years, the amplicon sequencing approach, especially those targeting mitochondrion DNA, has been increasingly used to trace the diversity of free-moving eukaryotic species in large open areas, such as the fish diversity in aquatic ecosystems (13–17). Comparatively, the more complex shotgun metagenomic data is in general used to profile prokaryotic communities and is rarely used for eukaryotic detection and profiling, mainly due to the lack of bioinformatic methodologies. Compared to amplicon sequencing strategies, shotgun metagenomic sequencing captures DNA fragments from all organisms present in the environments, including eukaryotes, and can bypass potential issues associated with polymerase chain reaction (PCR) amplification and primer biases (9, 12). Recently, several bioinformatics pipelines have been developed to detect eukaryotic DNA fragments in shotgun metagenomes, either based on reads or contigs (18, 19). For example, EukRep (20), Tiara (11), and EukDetect (12) are dedicated to improving the quality of eukaryotic genome reconstruction from environmental metagenomes (2, 18). CCMetagen (ConClave-based Metagenomics) (9) incorporates KMA (k-mer alignment) software (21) with the aim of including eukaryotes in studies of the metagenome. The development of these approaches provides alternative routines to trace eukaryotic diversity in complex environments via shotgun metagenomes, broadening the value of the high-cost shotgun metagenome data sets.

Associated with eukaryotic communities, a critical property is that they usually follow typical spatial and temporal ecological patterns (22). These include the well-recognized taxa-area relationship (TAR) and distance-decay relationship (DDR) at the spatial scale, and taxa-time relationship (TTR) and time-decay relationship (TDR) by extending spatial to temporal (23). Specifically, TAR and TTR describe the rate of increasing number of taxa along the increasing sampling area and observation time, respectively (24–27). DDR and TDR respectively reflect the decreasing pattern in community similarity with geographic distance and observation time intervals (27–29). Underlying these ecological patterns are multiple ecological processes, including deterministic and stochastic processes (30, 31). Characterizing these well-recognized ecological patterns and the underlying mechanisms not only reveals how the biological communities change across space and through time, but also serves as ecological indicators for assessing the reliability of the obtained community profiles.

The intertidal zone, located at the interface between terrestrial and marine ecosystems and exhibiting characteristics of both, is one of the most extensive and ecologically significant ecosystems (32, 33). With high environmental heterogeneity and dynamic changes in conditions, it is a valuable ecological entity and rich in biodiversity (32). Eukaryotes in the intertidal zone play important roles in nutrient cycling and energy metabolism, establishing complex food webs. Compared to prokaryotes, our ability to trace and profile intertidal non-fungal eukaryotic communities is no better or even worse than prokaryotes (34, 35). Therefore, the intertidal ecosystem can be used as an example system for biodiversity studies. Here, we aimed to address the following methodological and ecological questions: (i) How do the performances of different bioinformatic pipelines differ in detecting non-fungal eukaryotic DNA fragments from shotgun metagenomes? (ii) Do the recovered eukaryotic communities follow typical temporal patterns, for example, TTR and TDR? (iii) How does the shotgun metagenomic approach compare to 18S amplicon sequencing results in recovering eukaryotic information? By comparing the performances of different bioinformatic approaches, we presented an integrative approach to recover and trace non-fungal eukaryotic DNA fragments in complex shotgun metagenomes. We showed that the recovered eukaryotic communities from shotgun metagenomes also followed typical temporal patterns (e.g., TTR and TDR). Compared to 18S rRNA gene amplicon sequencing, the presented shotgun metagenome-based approach recovered higher eukaryotic diversity in the mudflat intertidal ecosystem with comparable temporal patterns. This study presents an alternative route to recover and trace eukaryotic DNA fragments in complex natural ecosystems and expands the conventional use of shotgun metagenomes from prokaryotes to eukaryotes.

## MATERIALS AND METHODS

### Location description and sample collection

Sedimental samples in this study were collected in a typical intertidal zone of Qingdao, China (36.46°N, 120.75°E), from August 2020 to June 2021 in a time series (every 2 months). Fifteen sediment samples were collected at each time point, six of which were sequenced with shotgun metagenomics. For each sample, five sediment cores (0–15 cm) were collected within 1 m^2^ and then evenly homogenized. The samples were then transported to the laboratory immediately on ice. Samples were stored at −80°C for DNA extraction and environmental parameter measurement.

### Measurement for environmental factors

Ten environmental factors were determined: temperature, salinity, pH, ammonia nitrogen (NH_4_^+^-N), nitrate nitrogen (NO_3_^−^-N), nitrite nitrogen (NO_2_^−^-N), total nitrogen (TN), total phosphorus (TP), sulfate (SO_4_^2−^) and total organic carbon (TOC). The temperature of the sediments was measured *in situ* using a mercury thermometer. Salinity was determined by a salinity meter (WS-31, Xudu, Beijing, China). The pH was measured with a pH meter (STARTER 300, OHAUS, Beijing, China). The TOC was determined by TOC-L CPH analyzer (Shimadzu, Kyoto, Japan). Concentrations of NH_4_^+^-N, NO_3_^−^-N, and NO_2_^−^-N in soil were determined through potassium chloride solution extraction followed by spectrophotometric analysis, with absorbance measured at 630 nm (GB/T7479-1987), 543 nm (GB7493-1987), and 410 nm (GB/T7487-1987), respectively (36). TN was quantified using the alkaline potassium persulfate digestion-UV spectrophotometric method (GB/T11894-1989), and TP was measured via the ammonium molybdate spectrophotometric method (GB/T11893-1989) (36). The SO_4_^2−^ concentration was analyzed using barium chromate spectrophotometry (GB/T11899-1989).

### DNA extraction, PCR amplification, and high-throughput sequencing

Total DNA was extracted from 0.5 g homogeneous sediment sample using a FastDNA SPIN Kit for Soil (MP Biomedicals, USA) according to the manufacturer’s manual. DNA quality was assessed by 260/280 and 260/230 nm ratios using a NanoDrop ONE Spectrophotometer (NanoDrop Technologies, Inc., Wilmington, DE, USA), and purified DNA was stored at −80°C until sequencing. Six randomly selected samples per time point were subjected to shotgun metagenome sequencing. Metagenomic reads were generated at 2 × 150 bp cycles on the Illumina NovaSeq 6000 platform (Illumina, Inc., San Diego, CA, USA). For each sample, the sequence data size ranged from 20.3 to 28.2 Gb, with an average data size of 22.4 Gb. The samples in each data set were barcoded, pooled in equimolar ratios, and sequenced concurrently in a single run without library replication. The high-throughput shotgun metagenomic sequencing was performed via NovoGene Co., Ltd. (Tianjin, China).

The 18S rRNA gene amplicon sequencing was performed in parallel to shotgun metagenomics to enable direct comparison of eukaryotic community composition between amplicon and metagenomic sequencing approaches. The V4 region of the eukaryotic 18S rRNA gene was amplified using the universal primer pairs 18S-528F/18S-706R (18S-528F, 5′-GCGGTAATTCCAGCTCCAA-3′; 18S-706R, 5′-AATCCRAGAATTTCACCTCT-3′) (37). To minimize potential additional bias, we used a two-step PCR amplification method (38). First, in triplicate, 10 ng of DNA from each sample was PCR-amplified in a 50 µL reaction followed by library enrichment with 10 cycles, using primers but no adaptors. The PCR amplicons were then purified and reconstituted in 50 µL of deionized water. Second, the purified PCR products (15 µL from each sample) were used in a new amplification step, also performed in triplicate. This stage involved primers integrated with adaptors, barcodes, and spacers, and the amplification was extended for 20 additional cycles. PCR amplification was carried out in a 25 µL reaction mix with 2.5 µL 10× AccuPrime PCR buffer (including dNTPs) (Invitrogen, Grand Island, NY, USA), 0.4 µM of each primer, 10 ng template DNA, and 0.2 µL Phusion DNA High-Fidelity polymerase (NEB). The cycling conditions comprised an initial denaturation at 94°C for 1 min, followed by 30 cycles of 94°C for 20 s, 62°C for 25 s, 68°C for 45 s, and a final extension at 68°C for 10 min. The amplicon sequencing of 18S rRNA genes was performed on the Illumina MiSeq PE250 platform (Illumina, Inc.) at Magigene Biotechnology Co., Ltd. (Guangzhou, China). On average, 83,201 reads per sample were obtained (Table S1).

### *De novo* assembly

Raw reads were input to Trimmomatic (v0.39) using default parameters, by which adaptors were removed and low-quality reads were filtered to yield high-quality reads (39, 40). The clean reads of sediment samples at each time point were co-assembled using MEGAHIT (v1.2.9) with a series of different k-mer sizes (-k-min 27 -k-max 127 -k-step 10), a strategy designed to reduce the number of true low-depth edges that were incorrectly identified as tips and removed by the cleaning algorithms (41).

### An integrative workflow for recovering eukaryotic sequences in metagenomes

Both contig-based (EukRep and Tiara) and read-based (CCMetagen) approaches were employed and assessed to identify eukaryotic sequences in metagenomes. For each approach, the recommended default thresholds were used. For contig-based analysis, the eukaryotic contigs derived from EukRep and Tiara were first dereplicated using SeqKit (v2.4.0) (42). Eukaryotic read mapping was performed via the CoverM approach (v0.6.1) (https://github.com/wwood/CoverM) (parameters: identity ≥ 95%, coverage ≥ 90%, “count” mode). The BAM files containing the mapping information of eukaryotic reads to contigs were obtained. Using SAMtools (v1.9) (43) and SeqKit (v2.4.0) (42), eukaryotic reads and taxonomic ids were extracted from bam files. The identified eukaryotic contigs were annotated by Kraken2 (v2.1.2) (44) and MEGAN (v6.24.20) (45) for taxonomic assignment at species level based on the NCBI NT database (https://ftp.ncbi.nlm.nih.gov/blast/db/FASTA/). Because MEGAN returned far fewer taxonomic assignments than Kraken2, only Kraken2 was used for further classification. For read-based analysis, the raw reads were mapped to the NCBI’s ncbi_nt_no_env_11jun2019 database (https://github.com/vrmarcelino/CCMetagen) using KMA (v1.4.3) (21), and then the KMA results were processed using CCMetagen to obtain eukaryotic reads and annotation. SeqKit was used to dereplicate and merge the eukaryotic reads obtained from the three abovementioned pipelines to obtain a comprehensive data set of eukaryotic reads. Finally, we integrated the annotation results obtained from Kraken2 and CCMetagen and employed TaxonKit (v0.15.1) (46) and Csvtk (v0.29.0) (47) to standardize the taxonomic names as NCBI.

### Amplicon sequencing data processing and statistical analyses

Raw amplicon FASTQ files were processed using the DADA2 (v1.26.0) package (48), including quality filtering, paired reads merging, chimeric removal, and amplicon sequencing variant (ASV) calling. Taxonomic assignment of eukaryotic ASVs was carried out using the PR2 database (v5.0.0) (49). Random subsampling of all samples to the same sequencing depth was carried out using the “Rarefy” function in the “GUniFrac” (v1.8) package. Fungal sequences were excluded from the obtained taxonomic profiles of metagenomic and amplicon sequencing considering the main focus of non-fungal eukaryotes in this study.

For eukaryotic recovery from shotgun metagenomes, the taxonomic classification performance of different methods was cross-evaluated with Kraken2 and visualized by non-metric multi-dimensional scaling (NMDS) ordination analyses. The profile table was normalized to the same read number (1,000 million) and average read length of 150 bp for each sample. The number of overlapping eukaryotic sequences identified by different methods was averaged across 36 samples and the proportion was calculated using the “eulerr” (v7.0.2) package in R (v4.4.0) (50). The composition of eukaryotic relative abundance profiles at the phylum and genus level was visualized using the “pcutils” (https://github.com/cran/pcutils.git) (v0.2.7) package in R. NMDS ordination was performed using the “metaMDS” function, with permutational multivariate analysis of variance (PERMANOVA) using the “adonis2” function in the “vegan” (v2.6-4) package (51) in R (50). The α-diversity (richness and Pielou evenness) and β-diversity (Bray-Curtis dissimilarity) were calculated using the “amplicon” (v1.19.0) and “vegan” (v2.6-4) packages (51, 52). The statistical significance of α-diversity metrics between metagenomic approaches and amplicon sequencing for 18S rRNA genes was assessed using the Mann-Whitney *U*-test (Wilcoxon test) in R, a non-parametric approach suitable for assessing differences in diversity indices. The Spearman correlation coefficient was calculated using the “quickcor” function from the “ggcor” (v0.9.8.1) package. Environmental heterogeneity was calculated between different samples based on normalized environmental factors using the Euclidean distance. To sort out the relative importance of deterministic and stochastic processes in eukaryotic community assembly, the “NST” (v3.1.10) package was employed to calculate the stochastic ratios (53). A total of 1000 null models were generated for the analyses. Stochastic ratios at each time point were calculated using taxonomic metrics based on the Bray-Curtis distance.

### Temporal pattern analyses

Two typical temporal patterns in ecology were examined, including TDR and TTR. We used linear regression in logarithmic space to test the strength of TDR, representing the relationship between eukaryotic compositional similarity and temporal distance among samples (54). Similar to the DDR (28, 55, 56), the temporal turnover of TDR was calculated as a similar linear least squares regression relationship between temporal distance and community similarity (based on the 1 − [Bray-Curtis dissimilarity in distance metric]). To model the relationship between species and time, we used the Arrhenius (log-log) plot with the formula: ln(*S_s_*) = c − *w*ln(*T*), where *S_s_* is the pairwise similarity of community composition, *T* is the time interval, the slope *w* is the TDR value, a measure of the temporal turnover of the community over time, and *c* is the constant. Both primary and quadratic linear fits were assessed.

TTR is conceptually an extension of TAR at the temporal scale (24, 25). The equation is commonly used to describe TTR: *S* = c*T*ω, where *S* is the species richness of the community, *T* is the observation time, *c* is the constant, and *ω* is the TTR value, which is a scaling index representing species turnover (24, 57). The slope coefficients of TTR were fitted by linear regression in logarithmic space to reflect observed community richness over the time span. We then also extended TDR and TTR to Hill numbers to assess the contribution of rare species (relative abundance <0.1%) to the temporal patterns. The α-diversity and β-diversity indices were calculated at different diversity orders *q* using the “hillR” (v0.5.2) package (https://github.com/daijiang/hillR) (58). All analyses were done in R (v4.4.0) (50).

## RESULTS

### Comparative evaluation of different approaches

**Fig 1:**
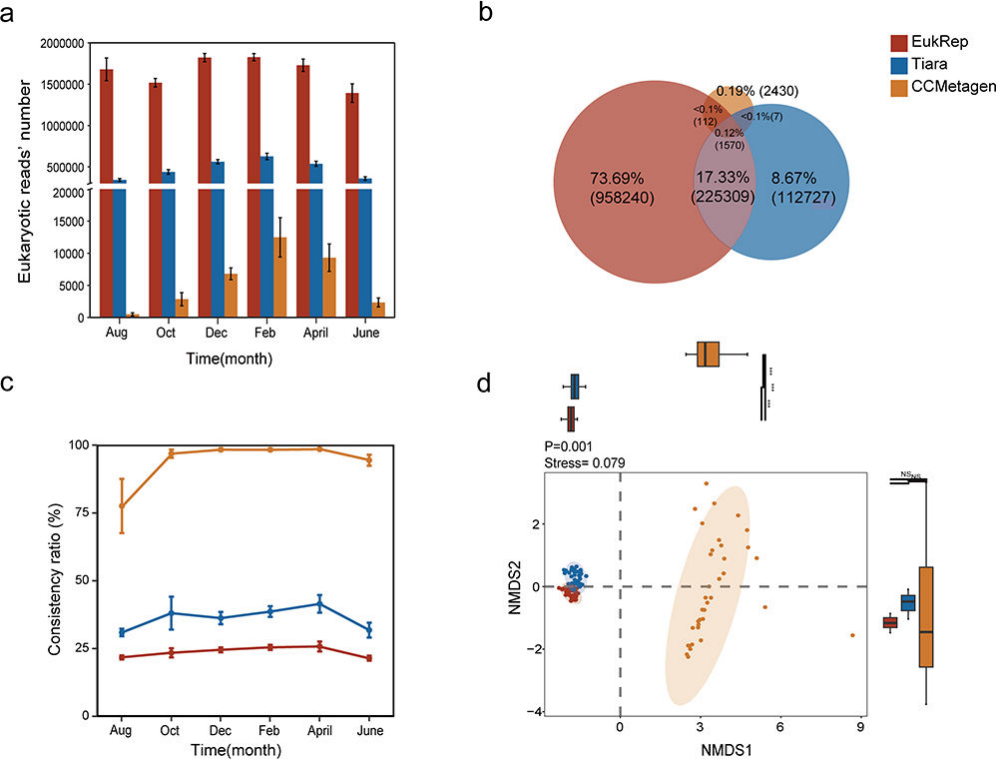
Comparative evaluation of different approaches in recovering eukaryotic communities from shotgun metagenomes. Three different approaches were evaluated, including EukRep, Tiara, and CCMetagen. (**a**) The number of recovered eukaryotic reads for different sampling time points. (**b**) The overlaps of identified eukaryotic sequences among different approaches. An average ratio across 36 samples was calculated for the overlap. Circle sizes were adjusted for clarity, as strict proportionality would render the CCMetagen circle too small to visualize. (**c**) The consistency of taxonomic assignments by different approaches with Kraken2. (**d**) The differences in eukaryotic communities recovered by different approaches at the species level were visualized at two-dimensional space by non-metric multi-dimensional scaling (NMDS) ordination analyses. The boxplots were included to provide additional visualization of the distribution of the samples within each group, complementing the NMDS ordination by highlighting group differences.

Multiple approaches have been developed to identify eukaryotic sequences in shotgun metagenomes, such as EukRep, Tiara, and CCMetagen, which were evaluated in this study. Of these, CCMetagen is read-based, and EukRep and Tiara are contig-based. More specifically, EukRep (v0.6.7) (20) and Tiara (v1.0.3) (11) use a k-mer-based approach to identify eukaryotic contigs. And CCMetagen (v1.4.1) (9) uses the ConClave (a scoring scheme within the KMA software) sorting-based approach that relies on the reference sequences to identify eukaryotic sequences directly from metagenomic reads (21). We first assessed these different tools using an artificial data set containing 95 eukaryotic and 308 bacterial species. As a result, EukRep and Tiara were found to have very high accuracy (94.47% and 92.78%) in identifying known eukaryotic contigs, whereas the performance of CCMetagen was quite low, though it achieved high precision (>99.78% across all taxonomic assignments) with minimal false positives (Fig. S2; Table S2). This was quite consistent with previous reports (59, 60). Taking the real-case evaluation below into account, this also suggested that the artificial data set cannot represent the diversity of real metagenomes well. For real-case evaluation, a data set containing 36 metagenomic samples collected over a 10-month period was analyzed (Fig. 1a). Prior to further detailed analyses, the shotgun metagenomic data set was assembled by MEGAHIT, generating a large volume of contigs, satisfying the requirement of contig-based approaches. As a result, EukRep was found to identify the most potential eukaryotic DNA sequences in the intertidal metagenomes. Using CCMetagen, metagenomic reads were directly mined for eukaryotic DNA fragments. On average, 4,007 reads were identified to be eukaryotic per sample (Table S3). For EukRep and Tiara, eukaryotic sequences were identified from assembled contigs. On average, 4,761 and 1,682 contigs were identified to be eukaryotic by EukRep and Tiara, respectively (Table S4). Then, reads were mapped to the eukaryotic contigs to generate read-level statistics. For EukRep and Tiara, the average number of mapped eukaryotic reads was respectively 1,183,660 (1.60%) and 339,612 (0.46%) (Table S3). We then looked into the overlaps of identified eukaryotic sequences among different approaches (Fig. 1b). Surprisingly low overlaps were observed among them, especially between CCMetagen and the other two approaches. The overlaps between EukRep and Tiara were relatively high in that 118,515 to 395,751 of identified eukaryotic sequences by Tiara were also detected by EukRep (Table S3). In contrast, as low as 5–5,097 eukaryotic sequences identified by CCMetagen were detected by EukRep, and 5–4,440 by Tiara (Table S3).

Notably, these different approaches are quite different in assigning the taxonomic information to eukaryotic sequences, with EukRep and Tiara not being able to provide species-level information. Here, we evaluated the consistency of taxonomic assignments from different approaches by comparing them with Kraken2 results (Fig. 1c). Of these, the average overlapping ratio between EukRep and Kraken2 was 23.71% (Table S3). Tiara was consistently found with higher ratios than EukRep, showing 36.17% overlaps with Kraken2 across different samples. In contrast, CCMetagen identified the least eukaryotic sequences, though many of these (42.62–100.00%) were verified by Kraken2. Second, the taxonomic compositions of the recovered eukaryotic communities were compared at the phylum level. In terms of the number of phyla obtained, EukRep had the most with 29 phyla, Tiara had 25 phyla, and CCMetagen had the least with 20 phyla. The composition of the eukaryotic communities recovered by EukRep and Tiara was highly similar at the phylum level, with Arthropoda (52.41% and 53.17%), Chordata (28.50% and 27.14%), Streptophyta (4.94% and 5.09%), and Bacillariophyta (3.83% and 3.51%) as the major taxa, and smoother changes over time (Fig. S1a and b). CCMetagen, on the other hand, differed from the former two in that the composition of the recovered eukaryotic community was relatively more similar to the amplicon method at the phylum level, with Bacillariophyta (61.85%), Annelida (12.39%), and Arthropoda (4.97%) as the major taxa, and a clearer trend in varying taxon abundance over time (Fig. S1c). Third, we also analyzed how the community similarity differed from each other using NMDS ordination analyses based on the eukaryotic community profile at the species level (Fig. 1d), showing significant differences by PERMANOVA with 999 permutations (*P* = 0.001). The communities obtained by EukRep and Tiara were very similar, while CCMetagen differed considerably from the other two approaches. Such results suggested highly differentiated eukaryotic community profiles identified by contig-based and read-based approaches, including the numbers, sequences, and composition.

### An integrative workflow to identify eukaryotic sequences in metagenomes

**Fig 2:**
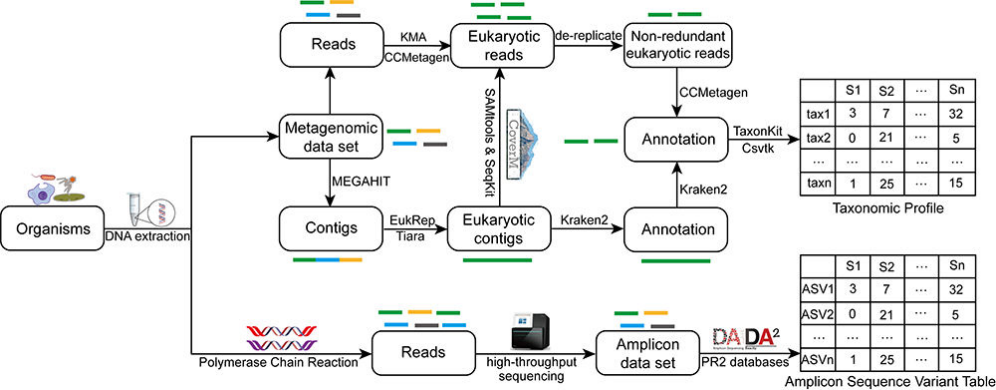
An integrative flowchart combining different approaches for recovering eukaryotic sequences from shotgun metagenomes. Total DNA extracted from sediment samples was subjected to shotgun metagenomic sequencing. The obtained metagenomic data were processed through multiple approaches to recover eukaryotic sequences: (i) read-based analysis, where raw reads were first mapped to reference databases using KMA (k-mer alignment) and subsequently classified by CCMetagen to identify eukaryotic reads; and (ii) contig-based analysis using EukRep and Tiara of metagenome-assembled contigs generated by MEGAHIT. The identified eukaryotic contigs were deduplicated and taxonomically annotated using Kraken2. The CoverM was used to generate the eukaryotic read mapping statistics, followed by extraction of the non-redundant eukaryotic reads using SAMtools and SeqKit. The extracted reads were merged with CCMetagen-derived reads. In parallel, 18S rRNA genes were amplified from extracted DNA, sequenced, and processed through the DADA2 pipeline, enabling comparative analysis of eukaryotic communities recovered by shotgun metagenomic and amplicon-based approaches.

Considering the vast differences between these approaches, here we proposed an integrative workflow to more comprehensively recover eukaryotic sequences in shotgun metagenomes (Fig. 2). In the workflow, three different approaches were integrated to identify eukaryotic sequences and obtain community profiles from shotgun metagenomes. First, shotgun metagenomic reads were directly parsed by CCMetagen to identify eukaryotic reads. Second, metagenomic data sets were first assembled into contigs, which were then analyzed by EukRep and Tiara to identify eukaryotic contigs. The reads were then mapped to the identified eukaryotic contigs. The mapped reads were then extracted and combined with the ones identified by CCMetagen. These reads were further dereplicated, forming a non-redundant eukaryotic data set of reads. Finally, the Kraken2 program was employed to assign taxonomic information to the identified eukaryotic contigs. By excluding fungal assignments, non-fungal eukaryotic community profiles were generated at different taxonomic levels. In addition, we also comparatively analyzed an amplicon data set targeting the 18S rRNA gene of eukaryotes, aiming to gain insights into how the shotgun metagenome-derived eukaryotic profiles differed from amplicon-based ones.

### Temporal dynamics of eukaryotic communities in mudflat intertidal zones

**Fig 3:**
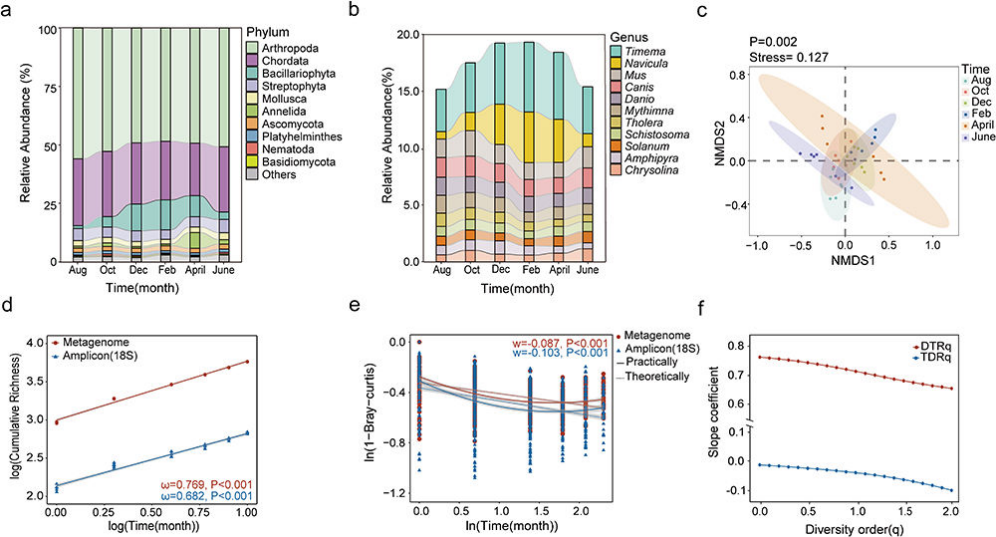
The composition and temporal patterns of the recovered non-fungal eukaryotic communities. (**a and b**) The taxonomic composition of the recovered non-fungal eukaryotic communities at the phylum and genus levels. (**c**) NMDS ordination analyses of non-fungal eukaryotic communities in different sampling months. (**d**) TTR analyses of the recovered eukaryotic communities based on shotgun metagenomes and 18S rRNA gene amplicon sequencing. (**e**) TDR analyses of the recovered eukaryotic communities based on shotgun metagenomes and 18S rRNA gene amplicon sequencing. (**f**) Extension of the diversity indices to Hill numbers for TTR and TDR at different diversity orders *q*. The higher the value of *q* is, the less weight is given to rare taxa.

By employing the above-described integrative workflow, the temporal dynamics of non-fungal eukaryotic communities in the mudflat intertidal zones were investigated. First, we analyzed the composition and dynamics of non-fungal eukaryotic communities along the sampling time point. Taxonomically, the intertidal eukaryotic communities were dominated by Arthropoda (59.55%), Chordata (24.06%), Bacillariophyta (8.32%), and Streptophyta (3.86%) at the phylum level (Fig. 3a), and by *Timema* (4.95%), *Navicula* (2.53%), *Mus* (1.70%), and *Canis* (1.64%) at the genus level (Fig. 3b). Second, clear seasonal patterns in relative abundance changes were observed for multiple eukaryotic groups. For instance, at the phylum level, the relative abundance of Bacillariophyta showed a seasonal trend of increasing in winter and then decreasing in summer, while Annelida had the highest relative abundance in April (Fig. 3a). At the genus level, the relative abundance of *Timema* and *Navicula* both showed a clear seasonal dynamic pattern of increasing in winter and then decreasing in summer (Fig. 3b). Third, clear seasonal patterns were also observed for the compositions of non-fungal eukaryotic communities (Fig. 3c). NMDS ordination analyses of these samples revealed that the community composition in August was more similar to that of October and the following June, and more different from that of December and the following April (PERMANOVA, *P* = 0.002). The differences in community composition between April and June of the following year were also greater. Samples collected in autumn and winter were more aggregated and had more similar community compositions, whereas samples collected in spring and summer were more dispersed and had more dissimilar community compositions. Fourth, TTR, which describes the accumulative pattern of eukaryotic taxa over the observation time, was investigated (Fig. 3d). The result revealed a strong eukaryotic TTR turnover ratio (*ω* = 0.769, *R*^2^ = 0.99, *P* < 0.001), as calculated as the slope of a linear regression fitting of the log-transformed taxa number and time. This suggested profound temporal turnovers for eukaryotic taxa in mudflat intertidal zones. Finally, we looked at TDR, which describes the decreasing pattern in community similarity with increasing time intervals. To do so, the eukaryotic community similarity was calculated based on Bray-Curtis dissimilarity, then TDR was assessed through a linear regression between log-transformed community similarity and time interval. As a result, although a significant TDR turnover ratio (*w* = −0.087, *R*^2^ = 0.20, *P* < 0.001) could be observed, the best fitting between community similarity and time interval was non-linear (Fig. 3e). Specifically, a trend of recovery in community similarity could be observed with more similar seasons, suggesting that seasonal variations imposed important roles in structuring intertidal eukaryotic communities. We also extended α-diversity and β-diversity to Hill numbers to analyze TTR and TDR patterns at different diversity orders (*q*) (Fig. 3f). Hill numbers, also known as effective numbers of species, are the integration of species richness and relative abundances into one class of diversity measures (61). The ambiguous definitions of abundant and rare taxa are resolved by setting different values of *q* (here 0 ≤ *q* ≤ 2). The weight given to rare taxa decreases with increasing *q* values. The slope coefficients of diversity-time relationships (DTR*_q_*) and TDRs (TDR*_q_*) decreased clearly with increasing *q* values from 0 to 2. This suggested that rare subcommunities played important roles in influencing the temporal patterns of intertidal eukaryotes. The rare taxa associated here were highly diverse, with genera such as *Anopheles cruzii* (0.1%), *Dioscorea cayenensis* (0.99%), and *Griposia aprilina* (0.1%) being dominant. Recent studies suggest that the relative abundance of rare subcommunities may be disproportionate to the degree of activity in ecosystem functioning and more vulnerable to environmental fluctuations (62–64). These results demonstrated that the intertidal eukaryotic communities recovered from shotgun metagenome sequences also followed typical ecological patterns, which also indicated the reliability of the obtained community profiles.

### Comparison with amplicon sequencing for eukaryotic community analyses

**Fig 4:**
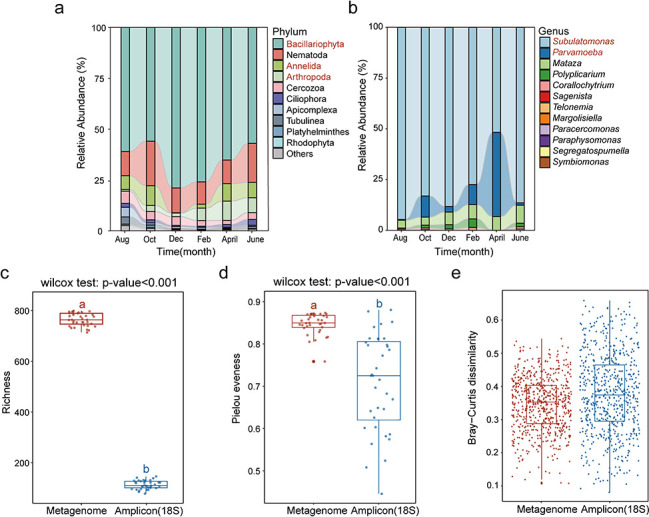
The composition and diversity indices of the recovered non-fungal eukaryotic communities in mudflat intertidal zones. (**a and b**) Phylum- and genus-level eukaryotic community composition obtained by amplicon sequencing of 18S rRNA genes. Red-colored phyla and genera indicated dramatic variations in relative abundance with time. (**c–e**) The diversity indices of recovered eukaryotic communities by shotgun metagenomes and 18S rRNA gene amplicon sequencing. Indices including richness, Pielou’s evenness, and Bray-Curtis dissimilarity were shown.

Amplicon sequencing of 18S rRNA gene PCR products is comparatively a more commonly used approach to profile micro-eukaryotic communities in complex natural environments. To gain a comparative view of how the eukaryotic communities profiled from shotgun metagenomes differed from the 18S rRNA gene profiles, comparative analyses were carried out. Taxonomically, a total of 36 eukaryotic phyla were obtained by the amplicon sequencing approach, slightly more than that by the workflow based on shotgun metagenomes. Of these, 15 eukaryotic phyla were detected by both approaches, but with highly differing relative abundances. Eukaryotic phyla including Bacillariophyta (62.04%), Nematoda (16.95%), Annelida (5.72%), and Arthropoda (5.02%) were found to be the dominant phyla in the amplicon sequencing data (Fig. 4a). Notably, the dynamic patterns of the relative abundances of Bacillariophyta and Arthropoda in both approaches were similar along the sampling points. At the genus level, amplicon sequencing of 18S rRNA genes tended to obtain fewer eukaryotic genera, of which *Subulatomonas* (80.28%), *Parvamoeba* (11.02%), and *Mataza* (6.10%) were the most dominant (Fig. 4b). Such results suggested dramatically different eukaryotic profiles recovered from different approaches.

Then, we looked into the diversity indices (e.g., α-diversity and β-diversity) and the patterns of the recovered eukaryotic communities by these two approaches. The genus-level eukaryotic community profiles were analyzed. As a result, significantly higher taxonomic richness (Mann-Whitney *U*-test, *P* = 3.02 × 10^−13^) and Pielou evenness (Mann-Whitney *U*-test, *P* = 3.11 × 10^−10^) were obtained by the metagenomic approach than those by the amplicon sequencing of the 18S rRNA gene (Fig. 4c and d). For β-diversity, comparable community dissimilarity was observed for these two different approaches (Fig. 4e). Consequently, similar temporal patterns including TDR and TTR were observed (Fig. 3d and e). A clear and steep temporal turnover ratio (*w* = −0.103) could be observed for the amplicon sequencing approach (Fig. 3e). Similarly, a high slope coefficient (*ω* = 0.682) was also observed for the TTR pattern of eukaryotic communities based on amplicon sequencing (Fig. 3d). Such results suggested that although dramatically different in eukaryotic profiles, the ecological patterns of the recovered eukaryotic communities revealed by these two approaches were comparable.

### Drivers of temporal dynamics of eukaryotic communities

**Fig 5:**
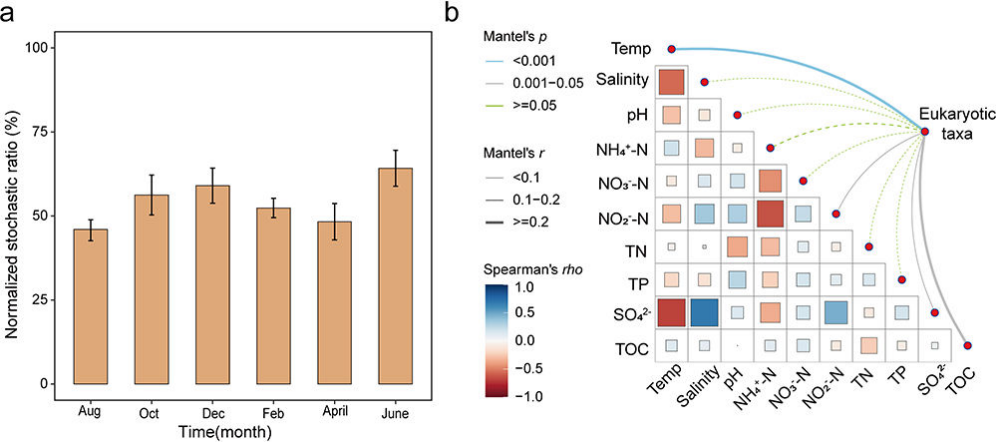
Ecological drivers of recovered non-fungal eukaryotic communities in mudflat intertides. (**a**) The stochastic ratios of recovered non-fungal eukaryotic communities were calculated for different sampling times. A total of 1,000 null models were generated. Stochastic ratios >50% indicate the dominance of stochastic processes. (**b**) Partial Mantel tests were used to determine the relationship between environmental factors and non-fungal eukaryotic communities. The edge colors represent Mantel’s *r* and *P* values, respectively, and the color gradient in the heatmap indicates Spearman’s correlation coefficients between different environmental factors.

It is commonly recognized that both deterministic and stochastic processes play important roles in community assembly, and that they together shape the compositional changes of biomes in complementary ways (53, 65). Here, we also assessed the ecological processes that mediated the temporal dynamics of intertidal eukaryotic communities. First, we quantified their relative importance in shaping the compositional variations of eukaryotic communities. Fluctuated stochastic ratios around 50% were observed, suggesting that the eukaryotic communities were more affected by stochastic processes from October to February and in June, while deterministic processes dominated in August and April (Fig. 5a). Second, a total of 10 environmental factors were measured, and their relative importance in driving the temporal dynamics of eukaryotic communities was explored by partial Mantel test. The results showed that changes in environmental factors were significantly associated with the compositional variations of eukaryotic communities (Mantel’s *r* = 0.374, *P* < 0.001). Among these, temperature, nitrite (NO_2_^−^-N), sulfate (SO_4_^2−^), and TOC were significantly correlated with the variations of eukaryotic communities (Fig. 5b), demonstrating the importance of these environmental parameters in structuring the temporal dynamics of intertidal eukaryotic communities.

## DISCUSSION

Shotgun metagenome sequencing has been routinely used in environmental biome studies, though it is associated with more complex data processing, analysis, interpretation, and higher cost compared to amplicon sequencing (66–70). In the past, the approach has mainly been used to profile complex prokaryotic communities in the environment, aiming to obtain the taxonomic composition and functional potential (71, 72). Notably, the extracted total DNA also contains genetic elements from eukaryotes, such as DNA fragments, microeukaryotes, and organismal residues. In most studies, the eukaryotic information carried by metagenomic data sets is usually ignored.

Several reasons may account for the insufficient mining of eukaryotic information from shotgun metagenomes. First, experimentally for environmental studies such as soil, only a small amount of sample is used for DNA extraction (e.g., 0.25–1 g) (73–75), excluding many visible eukaryotes. This makes typical shotgun metagenomic sequencing not a standardized approach for surveying eukaryotic communities. Second, currently developed bioinformatics approaches for metagenomes mainly target prokaryotic communities, for both taxonomic and functional profiles. Only a few approaches have been recently developed to specifically detect eukaryotic genetic sequences in shotgun metagenomes (9, 11, 12, 20). Third, compared to prokaryotes, much fewer genomes have been sequenced for eukaryotic species, further limiting metagenome-based tool development for eukaryotes. Meanwhile, eukaryotes generally have larger genomes and more complex genomic structures than prokaryotes, which makes the recovery of eukaryotic information from metagenomes more difficult and is not yet as commonly explored (2, 76, 77). Therefore, it is of necessity to evaluate and investigate the feasibility of recovering eukaryotic information from shotgun metagenomes.

In this study, we first comparatively investigated the differences between three recently developed approaches, including EukRep (20), Tiara (11), and CCMetagen (9), that hold the ability to recover eukaryotic sequences from shotgun metagenomes in a typical intertidal zone in Qingdao, China. Since these approaches differ from each other in the algorithms for detecting eukaryotic sequences, their results also differed clearly in our time-series metagenomic data set. At the algorithm level, EukRep and Tiara use k-mer counts to respectively identify eukaryotic contigs via support vector machines or neural networks (11, 20), while CCMetagen uses the ConClave classification scheme based on references to identify eukaryotic reads (9). Previous studies have demonstrated clear differences in the performance and accuracy of these approaches. EukRep, though capable of obtaining a large number of potential eukaryotic sequences, had poor precision and potentially high contamination, being consistent with previous results (11, 20, 77–79). CCMetagen, a reference-based method that uses the NCBI nucleotide collection as the reference database, therefore had relatively high precision among these three methods, but was lowest in the number of obtained eukaryotic sequences, mainly due to the low number of eukaryotic genomes in public databases (9). Comparatively, Tiara also did not perform clearly better than EukRep. Therefore, we proposed to integrate these different approaches to recover a more comprehensive eukaryotic data set from shotgun metagenomes, similar to the procedures in viral metagenomics (80). The integration of multiple approaches and the development of new methods are expected to improve eukaryote-centred metagenomics (81). Notably, the integrative approach here represents a working strategy but may not be the optimal choice for all studies, because incorporating different identification approaches and taxonomic assignment tools is expected to increase the recovered eukaryotic information in shotgun metagenomes. For instance, in the case fungi are targeted, employing marker gene-based tools such as 2bRAD-M by leveraging fungal databases tends to provide more accurate taxonomic information (82). In addition, integrating different sequencing technologies, such as next-generation and third-generation techniques, is also expected to improve eukaryotic sequence recovery (2, 78, 83).

The eukaryotic community compositions recovered from shotgun metagenomes were also compared with the ones derived from 18S rRNA gene amplicon sequencing. Although expected, dramatically different community compositions were observed. Arthropoda and Chordata were abundantly detected in shotgun metagenomes, while Bacillariophyta and Nematoda were the most abundant in the amplicon sequencing data. Compared with such a discrepancy between shotgun metagenomes and 16S rRNA gene amplicon sequencing observed for prokaryotic communities in some studies (84–87), the discrepancy for eukaryotic communities is surprisingly large. Multiple reasons may explain this striking scenario. First, technical differences should be the major reason. Shotgun metagenomics involves direct sequencing of isolated environmental DNA, whereas amplicon sequencing of the 18S rRNA genes relies on PCR amplification of specific regions using degenerated universal primers (88, 89). The shotgun metagenomic approach bypasses the PCR amplification step and avoids amplification biases, which are usually caused by primer mismatches relative to the target sequences and systematic differences in target regions (9, 12, 90). Usually, amplicon sequencing is biased to abundant species, resulting in higher differences in relative abundance for abundant and rare species (91–93). In contrast, shotgun metagenome sequencing is considered to be more accurate in quantifying microbial relative abundances (94–96). Second, the differences in genome size among eukaryotes are much larger than those among prokaryotes (97). Because large-sized genomes have higher chances to get sequenced and result in more sequences, this also eventually contributes to the observed discrepancy between shotgun metagenomes and amplicons. Third, the currently used PCR primers for 18S rRNA genes are usually used for micro-eukaryotes and may not well cover the wide diversity of eukaryotes (98, 99). All these abovementioned issues eventually lead to the large discrepancy between different technologies in recovering eukaryotic communities.

The shotgun metagenomic and amplicon sequencing data sets used in this study enabled us to comparatively investigate the temporal patterns of non-fungal eukaryotic communities in mudflat intertidal zones. Changes in the relative abundance and members of taxa over time were observed, with clear seasonal dynamics of eukaryotic communities along the sampling period. Consequently, both TTR and TDR were observed for the recovered eukaryotic communities, for both shotgun metagenomic and amplicon sequencing data sets. Of these, TTR demonstrates the rate of accumulatively observed species over the sampling period and is usually used as a macroecological metric of community dynamics (24–26). The temporal turnover represented by TDRs, which has been observed for both macrobes and microbes, is an important indicator of community succession (28, 54, 100–102). The observed temporal turnover of TDR is generally in line with the notions observed for other communities in different ecosystems (29, 54, 55, 103, 104). Previous studies suggest that changes in microbial communities over time are also influenced by other environmental factors, such as climate (28, 54). Here, environmental factors, including temperature, nitrite (NO_2_^−^-N), sulfate (SO_4_^2−^) and TOC, were found to be the most influencing. Further, by extending the diversity indices of TTR and TDR to Hill numbers, we found that rare subcommunities contributed significantly to the temporal patterns of eukaryotes in the intertidal zone. Although low in relative abundance, several studies have shown that they may participate in key functions disproportionate to their abundance (62, 105, 106). Here for non-fungal eukaryotes, some of these lowly abundant taxa also represented their footprints in the environment, and accumulative sampling effort was needed to capture them. Interestingly, some recent studies show the interdependence of spatial and temporal accumulation of species in species-time-area relationships in different systems (24, 107), resolving the turnover of biological communities in both spatial and temporal dimensions. Notably, the comparable turnover ratios observed for shotgun metagenomic and amplicon sequencing data sets suggested similar ecological patterns for the recovered non-fungal eukaryotic communities, though the recovered community profiles dramatically differed. This also suggested that these different technologies may be complementarily used to uncover the diversity patterns of non-fungal eukaryotic communities, as has been done for prokaryotic communities (24, 108–110).

In summary, this study comparatively investigated the performances of different computational approaches to identify non-fungal eukaryotic sequences from shotgun metagenomes in a typical intertidal zone in Qingdao, China. Since different approaches differed greatly in the recovered eukaryotic sequences, we proposed to integrate multiple methods to generate eukaryotic community profiles. We then employed the integrated approach to recover non-fungal eukaryotic community profiles from shotgun metagenomes along a temporal scale. Meanwhile, the eukaryotic community profiles generated from amplicon sequencing of 18S rRNA genes were also comparatively analyzed. Comparable temporal patterns were observed for the recovered non-fungal eukaryotic communities by both approaches, though the community compositions dramatically differed. This study demonstrates an alternative route in tracing non-fungal eukaryotic communities in complex environments and fosters wider applications of the shotgun metagenomes generated by researchers.

## Data Availability

Raw data of the mudflat intertidal metagenomes and 18S rDNA amplicons sequencing generated in this study have been deposited in the NCBI Sequence Read Archive (SRA) database under project IDs PRJNA957716 and PRJNA1029225. The R scripts developed for this study have been uploaded to github and can be downloaded from https://github.com/HeHan-hub/Tracing-non-fungal-eukaryotic-diversity-via-shotgun-metagenomes-in-the-complex-mudflat-intertidal.git.

## SUPPLEMENTAL MATERIAL

Supplemental material msystems.00413-25-s0001.pdf Fig. S1 and S2; captions for Tables S1 to S4.

Table S1 msystems.00413-25-s0002.xlsx Number of raw reads for amplicon sequencing of 18S rRNA genes per sample.

Table S2 msystems.00413-25-s0003.xlsx Benchmarking of different software with known data sets.

Table S3 msystems.00413-25-s0004.xlsx Number of eukaryotic reads recovered by different tools and overlaps.

Table S4 msystems.00413-25-s0005.xlsx Number of eukaryotic contigs recovered by different tools and overlaps.

ASM does not own the copyrights to Supplemental Material that may be linked to, or accessed through, an article. The authors have granted ASM a non-exclusive, world-wide license to publish the Supplemental Material files. Please contact the corresponding author directly for reuse.
