# Supplementary material for: Tracing non-fungal eukaryotic diversity via shotgun metagenomes in the complex mudflat intertidal zones
Source: mSystems. 2025 Jun 12;10(7):e00413-25. doi: 10.1128/msystems.00413-25 (PMC12282072; doi:10.1128/msystems.00413-25)
Supplement: Supplemental material — Fig. S1 and S2; captions for Tables S1 to S4. [file msystems.00413-25-s0001.pdf]

Tracing non-fungal eukaryotic diversity via shotgun metagenomes in the complex mudflat intertides

He Han[1], Mengzhi Ji[1], Yan Li[1], Xiaofan Gong[1], Wen Song[1], Jiayin Zhou[1], Kai Ma[1], Yuqi Zhou[1], Xia Liu[1], Mengqi Wang[1], Yueyue Li[1], Qichao Tu[1,2,*]

[1] Institute of Marine Science and Technology, Shandong University, Qingdao, China 266237

[2] Qingdao Key Laboratory of Ocean Carbon Sequestration and Negative Emission Technology, Shandong University, Qingdao, China

[*] Correspondence should be addressed to tuqichao@sdu.edu.cn

Tel: 86-0532-58633267; Fax: 86- 0532-58633218

Supplementary Figures

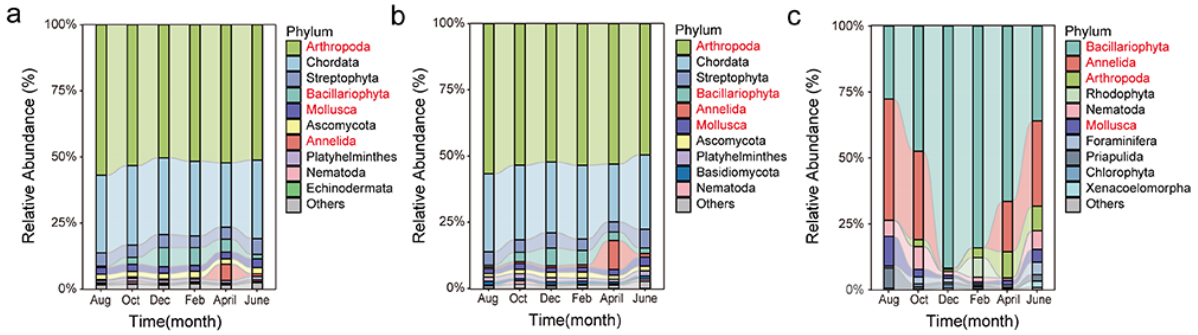

Supplementary Figure 1. The composition of the eukaryotic communities recovered by different approaches at the phylum level. (a-c) The composition of the eukaryotic communities recovered by EukRep, Tiara and CCMetagen at the phylum level. The red sections showed the same phylum obtained by the three methods.

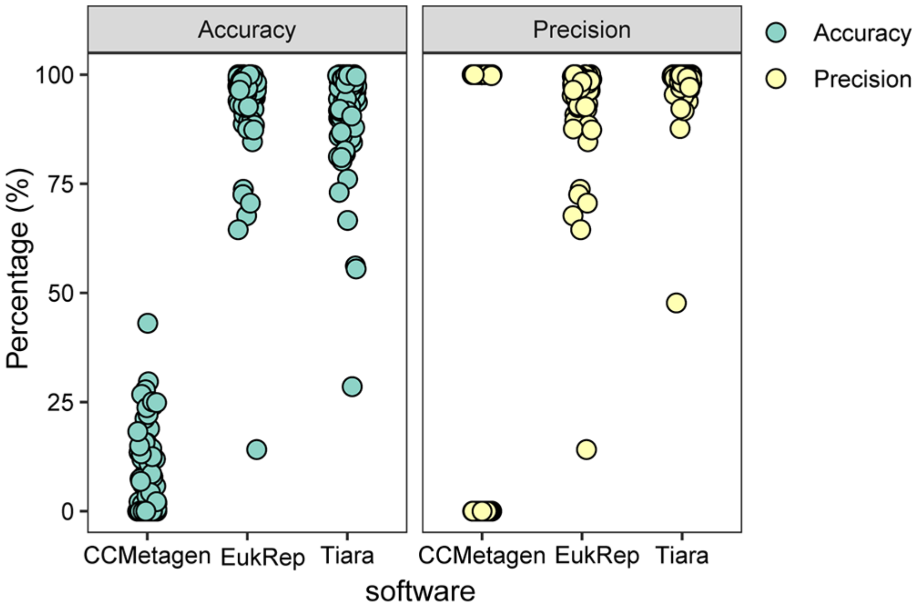

Supplementary Figure 2. Benchmarking performance of eukaryotic identification by different approaches on the simulated metagenomic dataset.

Supplementary Tables

Supplementary Table 1. Number of raw reads for amplicon sequencing of 18S rRNA genes per sample.

Supplementary Table 2. Benchmarking of different software with known datasets.

Supplementary Table 3. Number of eukaryotic reads recovered by different tools and overlaps.

Supplementary Table 4. Number of eukaryotic contigs recovered by different tools and overlaps. Percentages of consistency between different approaches and Kraken2 are also listed.
